# Midkine as a prognostic biomarker in oral squamous cell carcinoma

**DOI:** 10.1038/sj.bjc.6604539

**Published:** 2008-08-05

**Authors:** K Ota, H Fujimori, M Ueda, S Shiniriki, M Kudo, H Jono, Y Fukuyoshi, Y Yamamoto, H Sugiuchi, H Iwase, M Shinohara, Y Ando

**Affiliations:** 1 Department of Oral and Maxillofacial Surgery, Graduate School of Medical Sciences, Kumamoto University, 1-1-1 Honjo, Kumamoto 860-8556, Japan; 2 Department of Diagnostic Medicine, Graduate School of Medical Sciences, Kumamoto University, 1-1-1 Honjo, Kumamoto 860-8556, Japan; 3 Department of Neurosurgery, Graduate School of Medical Sciences, Kumamoto University, 1-1-1 Honjo, Kumamoto 860-8556, Japan; 4 Department of Breast and Endocrine Surgery, Graduate School of Medical Sciences, Kumamoto University, 1-1-1 Honjo, Kumamoto 860-8556, Japan; 5 Department of Medical Technology, Kumamoto Health Science University, 325 Izumi, Kumamoto 861-5598, Japan

**Keywords:** midkine, oral squamous cell carcinoma, biomarker, cancer screening, prognosis prediction

## Abstract

The aim of this study was to evaluate serum midkine (S-MK) concentrations as a prognostic tumour marker in oral squamous cell carcinoma (OSCC). We measured S-MK concentrations in patients with OSCC and healthy volunteers. In addition, we performed real-time quantitative reverse transcription–PCR analysis and immunohistochemistry with fresh tumour samples. To determine whether S-MK concentrations have prognostic value, we performed survival analyses with clinical information by using the log-rank test. Serum midkine concentrations were significantly higher in patients with OSCC than in healthy controls (*P*<0.001). Serum midkine concentrations were also significantly increased in early-stage OSCC compared with those of healthy individuals (*P*<0.001). In addition, immunohistochemistry allowed identification of overexpressed MK protein in OSCC tissues. MK mRNA showed higher expression in OSCC samples compared with normal mucosal samples. Patients in high S-MK groups showed a significantly lower 5-year survival rate compared with patients in low S-MK groups (*P*<0.05). The increased S-MK concentrations in early-stage OSCC were strongly associated with poor survival. Serum midkine concentrations may thus be a useful marker not only for cancer screening but also for predicting prognosis of OSCC patients.

Oral cancer is one of the common types of human cancer (Petersen, 2003; Jemal *et al*, 2006). The survival rate for patients with oral cancer has not yet improved, despite better diagnostic techniques and innovations in treatments. Improved survival of patients with oral cancer requires better techniques for the prediction of prognosis. Oral squamous cell carcinoma (OSCC), the most common of several types of oral cancers, does not have a good prognosis. Several researchers have studied the usefulness of tumour-associated antigens for primary diagnosis of OSCC, but no tumour markers have given a precise prediction of prognosis (Tsutsui *et al*, 1993; Krimmel *et al*, 1998).

Midkine (MK), a heparin-binding growth factor, is expressed intensely during the midgestation period and its expression becomes generally weak in adults (Kadomatsu *et al*, 1988; Muramatsu, 2002). Midkine has various biological activities such as neuronal survival, tissue repair, and carcinogenesis. Midkine, which has neuroprotective activity and neurite extension (Michikawa *et al*, 1993; Owada *et al*, 1999), expresses strongly in cerebral infarct (Yoshida *et al*, 1995) and Alzheimer's disease (Yasuhara *et al*, 1993; Salama *et al*, 2005). Midkine promotes the migration of inflammatory cells, specifically macrophages and neutrophils (Takada *et al*, 1997; Horiba *et al*, 2000). With these activities, MK expression increases at intraperitoneal adhesions after surgery (Inoh *et al*, 2004) and cardiac remodelling after myocardial infarction (Obama *et al*, 1998; Horiba *et al*, 2006). In addition, MK plays an important role in carcinogenesis. Midkine expression in various malignant tumours is higher than that in normal tissues (Garver *et al*, 1993, 1994; Konishi *et al*, 1999; Ye *et al*, 1999). Midkine protein expression was reported to be correlated with poor prognosis in patients with neuroblastomas (Nakagawara *et al*, 1995), astrocytomas (Mishima *et al*, 1997), pancreatic head carcinomas (Maeda *et al*, 2007), or gastrointestinal stromal tumours (Kaifi *et al*, 2007). In addition, because MK protein is a plasma-secreted protein, MK levels in blood may increase in patients with malignant diseases. Enzyme-linked immunoassay (EIA) has allowed measurement of MK levels in blood, and increased blood MK levels have been reported in patients with malignant tumours, including hepatocellular carcinoma (Muramatsu *et al*, 1996), gastric carcinoma, and lung carcinoma (Ikematsu *et al*, 2000). These reports suggest that MK in blood may have the potential to become a very useful tumour marker. Recent studies have found increased preoperative S-MK concentrations in patients with oesophageal squamous cell carcinoma (Shimada *et al*, 2003). In addition, higher plasma MK concentrations in patients with neuroblastoma were strongly correlated with poor survival (Ikematsu *et al*, 2003). Midkine levels in blood may thus become a useful tumour marker for predicting prognosis of cancer patients. However, no studies have focused on the correlation between S-MK expression and OSCC.

In this study, we evaluated clinicopathological data and analysed S-MK concentrations in 60 patients with OSCC and 134 healthy volunteers. We used a novel automated fluorescent-enzyme immunoassay (FEIA) system for this analysis. In addition, we compared the expression of MK protein in OSCC tissues and normal mucosal tissues by means of immunohistochemistry and real-time polymerase chain reaction (PCR).

## Materials and methods

### Patients and healthy volunteers

A total of 60 patients with primary OSCC who were treated at the Department of Oral and Maxillofacial Surgery (Kumamoto University Hospital), between 1999 and 2004 were studied. We excluded patients who presented with distant metastases and all patients who underwent radical treatments. Fifty-three patients had surgery with more than 10 mm resection margins and no microscopic residual tumour, and of those, 36 patients underwent preoperative chemoradiotherapy. Seven patients underwent radical chemoradiotherapy and we confirmed no residual tumour and no local recurrence. Of the 60 OSCC patients, 37 were males and 23 were females, with a mean age of 66.8±11.4 years (median 67; range 27–92). All patients were staged according to the 1997 UICC *TNM Classification of Malignant Tumors* (Sobin and Wittekind, 2002). Clinicopathological data, including age, sex, blood data (leukocyte count and platelet count), tumour size, nodal status (N factor; cervical lymph node metastasis), degree of differentiation of OSCC, and prognosis, were obtained from patient files. Serum samples were collected from all patients; biopsy tissue specimens were obtained from 52 patients before treatment.

We also collected samples from healthy volunteers who did not have any disorders, such as liver dysfunction and rheumatoid arthritis, after they underwent medical check-ups at Kumamoto University Hospital. In total, we evaluated 134 serum samples and 27 normal oral mucosal specimens, which we excised surgically when we extracted teeth. Of the 134 healthy volunteers, 73 were males and 61 were females, with a mean age of 63.8±11.0 years (median 65; range 20–92). Twenty-seven healthy volunteers (20.1%) and 16 OSCC patients (26.7%) had a smoking habit. Between OSCC patients and healthy volunteers, the smoking habit nearly matched. All subjects gave their informed consent.

All serum samples were collected, by means of venipuncture, from patients and volunteers after they gave informed consent. Venous blood was allowed to clot for 30 min at room temperature and was then centrifuged for 10 min at 3000 r.p.m. Serum samples were collected from OSCC patients before treatment. Serum samples and biopsy specimens were stored at –80°C until MK assays and real-time PCR. The remaining tumour materials were fixed in 10% formalin for immunohistochemistry before processing.

### EIA for MK

The EIA MK assay is a two-site immunoenzymometric assay, requiring 50 *μ*l of serum sample, which was performed automatically using an immunoassay analyser, AIA-600II (Tosoh Corporation, Tokyo, Japan). Midkine in a sample was simultaneously reacted with mouse anti-midkine monoclonal antibody (SC-2) immobilised on magnetisable beads and with alkaline phosphatase-labelled mouse anti-midkine monoclonal antibody (SC-4) to form a sandwich structure. After 10 min of incubation at 37°C, the beads were washed to remove unbound materials. A fluorogenic substrate, 4-methylumbelliferyl phosphate, was added for the enzyme-substrate reaction at 37°C for 5 min. The rate of fluorescence of converted 4-methylumbelliferone was directly proportional to the MK concentration in the samples.

### RNA isolation and real-time PCR

Total RNA was extracted by using the RNeasy Mini Kit (Qiagen, Hilden, Germany) and treated with the RNase-Free DNase Set (Qiagen) according to the manufacturer's instructions. Total RNA was quantified with a NanoDrop ND-1000 (NanoDrop Technologies, Wilmington, DE, USA) spectrophotometer and software program. Total RNA (0.5 *μ*g) in samples was reverse transcribed to complementary DNA (cDNA) by using the ExScript RT reagent (Takara Bio Inc., Otsu, Japan), according to the manufacturer's protocol. All PCR reactions were performed by using the LightCycler 480 System (Roche Diagnostics, Basel, Switzerland) with a LightCycler 480 SYBR Green I Master kit (Roche Diagnostics). A reaction mixture was added to 2.0 *μ*l of cDNA for each sample. The reaction mixture contained the following components: 6.0 *μ*l of water, 1 *μ*l of forward primer (0.5 *μ*M), 1 *μ*l of reverse primer (0.5 *μ*M), and 10 *μ*l of 2 × Master mix. The primers used for real-time PCR were as follows: *MK* forward 5′-AGATGCAGCACCGAGGCT-3′, *MK* reverse 5′-CTTTCTTTTTGGCGACCG-3′; *β_2_-microgloblin (β2M)* forward 5′-CGGGCATTCCTGAAGCTGA-3′, and *β2M* reverse 5′-GGATGGATGAAACCCAGACACATAG-3′.

The *β*2M gene was chosen for normalisation of data. Each reaction was performed under the following conditions: initialisation for 10 s at 95°C and then 45 cycles of amplification, with 5 s at 95°C for denaturation and 20 s at 60°C for annealing and elongation. After amplification, the temperature was slowly raised to above the melting temperature of the PCR product to measure fluorescence and thereby to determine the melting curve. In addition, to ensure RNA quality, several preparations were subjected to analysis of expression using the Hitachi SV1210 microchip electrophoresis system (Hitachi Electronics Engineering Company, Tokyo, Japan). A standard curve was plotted for each primer probe established by using serial dilution of pooled cDNA from tissues. All standards and samples were analysed in triplicate.

### Immunohistochemistry

To detect MK in OSCC samples, we performed immunohistochemical analysis with two monoclonal MK antibodies: IP-10, with an epitope in the N-terminal half of the MK fragment, and IP-14, reacting with the C-terminal half of the MK fragment. The primary antibodies IP-10 and IP-14 were generated by Cell Signals Inc. (Yokohama, Japan). Paraffin-embedded 4-*μ*m-thick sections were prepared, deparaffinised in xylene, and rehydrated in graded alcohols. Endogenous peroxidase activity was blocked by immersing sections in 0.3% hydrogen peroxide in methanol for 30 min, and antigen retrieval was performed by a 15-min microwave pretreatment in citrate buffer (pH 6.0, 0.01 mol l^−1^). After being incubated with Protein Block Serum-Free (Dako, Glostrup, Denmark), sections were incubated overnight at 4°C with mouse monoclonal antibodies (IP-10 at 1 : 25; IP-14 at 1 : 100). After incubation with antimouse labelled polymer (EnVision^+^ System HPR; Dako) for 30 min at room temperature, 3,3′-diaminobenzidine was used as the chromogen. Sections were immunohistochemically stained and were then counterstained with hematoxylin to enhance nuclear detection.

### Statistical analysis

The normality of the data was first assessed using the Shapiro–Wilks test. Data were then evaluated using Student's *t*-test and an analysis of variance (normal distributed data) or by the Kruskall–Wallis test (non-normal data). Regression analysis was done by simple regression on S-MK concentrations to leukocyte or platelet. To determine the cutoff point of S-MK concentrations, receiver-operating characteristic (ROC) curves were constructed by calculating the sensitivities and specificities for cutoff values. The optimal cutoff values were selected on the basis of the extreme upper left points of the ROC curves. Survival curves were plotted by using the Kaplan–Meier method and analysed with the log-rank test for univariate analysis. All analyses were performed with the JMP (version 5.0.1; SAS Institute Japan, Tokyo, Japan). *P*-values of less than 0.05 were regarded as statistically significant.

## Results

### S-MK concentration and its correlation with prognosis in OSCC

**Figure 1:**
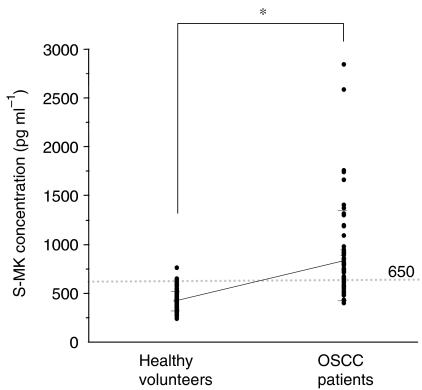
Serum midkine concentrations for healthy volunteers and OSCC patients. Healthy volunteers (*n*=134; 80 males and 54 females), and OSCC patients (*n*=60; 38 males and 22 females). Statistical significance was evaluated using Student's *t*-test. ^*^P<0.001.

**Figure 2:**
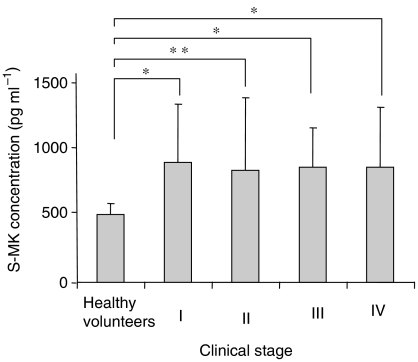
Serum midkine concentrations at each clinical stage in OSCC patients and in healthy volunteers. Statistical significances were evaluated using Student's *t*-test. ^*^P<0.001, ^**^P<0.01.

We determined S-MK concentrations in samples from 60 OSCC patients and 134 healthy volunteers. The mean (±s.d.) S-MK concentration was 885.9±465.0 pg ml^−1^ for the OSCC patients and 419.1±97.9 pg ml^−1^ for the healthy subjects. Serum midkine concentrations for the OSCC patients were significantly higher than those for healthy volunteers (Figure 1). To examine the usefulness of this marker protein, a cutoff point was determined for each set of values, by using a ROC curve, under the condition of specificity equal to 99.3%. With a cutoff value of 650, 66.7% of the OSCC patients had S-MK values that were positive predictive markers. As Table 1 shows, S-MK concentrations in patients and healthy subjects older than 60 years were significantly higher compared with those in patients and healthy subjects younger than 60 years, respectively. We compared S-MK concentrations in healthy volunteers and OSCC patients under 60 and over 60 years old. Serum midkine concentrations of OSCC patients were significantly higher than those in healthy volunteers over 60 years old (965.8±505.5 and 456.3±119.2 pg ml^−1^, respectively). Moreover, in the analysis of both groups under the age of 60 years old, the concentrations in OSCC patients were significantly higher than those in healthy volunteers (645.9±160.8 and 345.3±93.6 pg ml^−1^, respectively). We concluded from these analyses that, independently from the ages, S-MK concentrations in OSCC patients were significantly higher than those in age-matched healthy volunteers. We also studied S-MK concentrations in relation to clinicopathological parameters (sex, age, smoking habit, clinical stage, tumour size, cervical lymph node metastasis, degree of differentiation of OSCC, and prognosis) in OSCC patients (Tables 1 and 2). However, we found no correlation between S-MK and sex, tumour size, cervical lymph node metastasis, or degree of differentiation of OSCC. Although S-MK concentrations showed no increasing tendency related to clinical stage, concentrations were significantly elevated in all stages in OSCC compared with those in healthy volunteers (stage I, III, IV; *P*<0.001, stage II; *P*<0.01) (Figure 2). We measured leukocyte and platelet counts to examine the influence of S-MK concentrations. With simple regression analysis, a relationship was not found between S-MK concentrations and the numbers of leukocytes or platelets (leukocytes *r*=0.03 *P*=0.80; platelets *r*=0.02 *P*=0.90). We also measured the MK concentration in plasma in 60 OSCC patients and 91 healthy volunteers. Plasma MK (P-MK) concentrations in OSCC patients and healthy volunteers were 783.6±286.1 and 404.2±89.3 pg ml^−1^, respectively. Oral squamous cell carcinoma patients had significantly higher P-MK concentrations than did healthy volunteers (*P*<0.0001).

**Figure 3:**
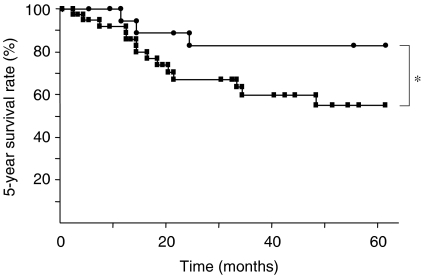
Survival of OSCC patients with high or low S-MK concentrations. Closed squares: patients with S-MK concentrations of ⩾650 pg ml^−1^ (*n*=40) had a survival rate of 56.6%. Closed circles: patients with S-MK concentrations of <650 pg ml^−1^ (*n*=20) had a survival rate of 82.9%. Statistical significances were evaluated using log-rank test. ^*^P<0.05.

In our investigation of the relationship between S-MK concentrations and prognosis, we divided OSCC S-MK concentrations into two groups: higher than or equal to 650 pg ml^−1^ (*n*=40) or lower than 650 pg ml^−1^ (*n*=20). The lower S-MK concentration group had a significantly greater 5-year survival rate than did the higher S-MK concentration group (82.9 *vs* 56.6%; *P*<0.05) (Figure 3). The median survival time of OSCC patients with high S-MK groups was 62 months, but the survival rate in low S-MK groups was not below 50%, whereas the average observation period after radical treatment was 69.2 months. We observed no significant relationship between 5-year survival rate and age. However, as Table 2 shows, clinical stage, tumour size, and cervical lymph node metastasis were significantly related to 5-year survival rate.

### MK mRNA levels in OSCC

**Figure 4:**
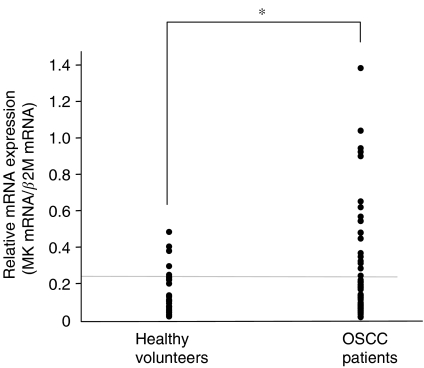
MK mRNA expression for healthy individuals and OSCC patients. Healthy volunteers (*n*=28) and OSCC patients (*n*=60) were subjected to this study. *β*_2_-Microgloblin was used to normalise the data as described in the text. Statistical significance was evaluated using Student's *t*-test. ^*^P<0.05.

To examine the relative expression of the *MK* gene in OSCC samples, we used real-time PCR to determine *MK* mRNA levels in 60 samples from OSCC patients and 28 samples from healthy volunteers. *Midkine* mRNA levels were significantly higher in OSCC tissues than in normal mucosal tissues (0.28 *vs* 0.15 *P*<0.05) (Figure 4). However, *MK* mRNA expression in each OSCC tissue specimen was not significantly associated with S-MK concentration (data not shown).

### Immunohistochemical staining of MK in OSCC

**Figure 5:**
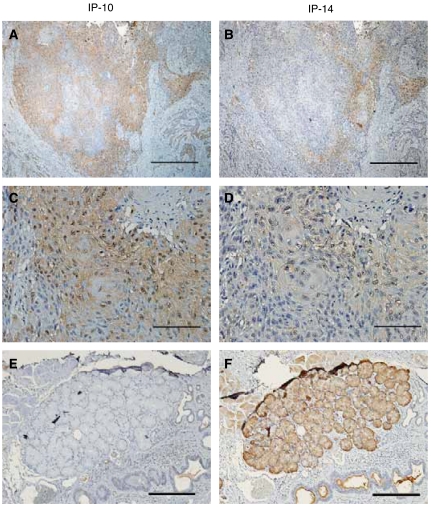
Immunohistochemistry with anti-MK antibody in OSCC and glandular duct samples. The biopsy specimen was obtained from a 59-year-old man with OSCC (T2N2bM0, stage IV). (**A**), (**C**), and (**E**) Immunohistochemistry with IP-10 antibody. (**B**), (**D**), and (**F**) immunohistochemistry with IP-14 antibody. (**A**–**D**) The OSCC tissues and (**E** and **F**) glandular ducts. (**A** and **B**) original magnification, × 40, scale bar=1 mm; (**C** and **D**) original magnification, × 200, scale bar=200 *μ*m; and (**E** and **F**) original magnification, × 100, scale bar=500 *μ*m.

Immunoreactive MK was localised in cancer cell cytoplasm. Out of 52 OSCC samples evaluated with IP-10 antibody, 45 were immunoreactive; with IP-14 antibody, 43 samples also showed a positive reaction. However, differences in reactivity – chromatic strength and localisation of staining – were noted with the IP-10 and IP-14 antibodies. Recognition of tumour cells by IP-10 was greater, and only IP-14 was immunoreactive with glandular duct (Figure 5).

## Discussion

**Figure 6:**
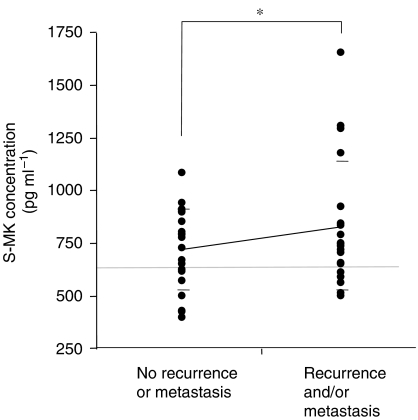
Serum midkine concentrations in the group with no recurrence or metastasis and the group with recurrence and/or metastasis. Statistical significance was evaluated using Student's *t*-test. ^*^P=0.17.

Our study produced two important findings. First, even though S-MK concentrations were not related to clinical stage, tumour size, and cervical lymph node metastasis, S-MK concentration offers promise as a novel tumour marker of prognosis in OSCC. Serum midkine concentrations will allow us to predict prognosis because, in the presence of highly elevated concentrations, we would use a larger surgically margin when removing a tumour and would perform chemotherapy and/or radiotherapy before or after the operation with better results. Midkine has been well documented to promote various activities related to oncogenesis and tumour progression, including cell migration (Takada *et al*, 1997), angiogenic functions (Choudhuri *et al*, 1997), mitogenesis (Muramatsu and Muramatsu, 1991), and antiapoptosis (Owada *et al*, 1999). Midkine can also give cancerous cells a growth advantage through antiapoptotic activity. Qi *et al* (2000) observed that MK protein rescued G401 cells, a Wilms’ tumour cell line, from cisplatin-induced apoptosis by upregulation of Bcl-2. Mirkin *et al* (2005) reported that increased MK expression exerted a significant cytoprotective effect against doxorubicin in drug-sensitive cells. They suggested that MK indirectly mediates acquired drug resistance to protect neighbouring drug-sensitive cells and contributes to the development of resistance to chemotherapeutics. In 40 patients who underwent an operation and chemotherapy, S-MK concentrations tended to be higher in the group with recurrence and/or metastasis than in the group without recurrence or metastasis (Figure 6). We confirmed the belief that MK directly or indirectly aids survival of cancer cells through antiapoptosis and provides cytoprotection against chemotherapy and radiotherapy. Takei *et al* (2006) reported that a combined therapy involving MK small interfering RNA and Paclitaxel significantly enhanced anticancer activity or maintained the effective anticancer activity of Paclitaxel. To inhibit the secretion of MK may be a novel therapy against drug-resistant cancers. In addition, MK protein expression in OSCC cases was reported to be significantly correlated with the expression of vascular endothelial growth factor (VEGF) (Ruan *et al*, 2007), and S-MK concentration was correlated with serum levels of VEGF-C (Krzystek-Korpacka *et al*, 2007). Expression of VEGF was also significantly correlated with prognosis in OSCC (Uehara *et al*, 2004). In these previous results, MK may be related to a prognosis through the activation of VEGF. However, it was reported that MK downregulated VEGF-A-induced neovascularisation and vascular permeability in a recent study (van der Horst *et al*, 2008). The involvement of MK in tumour angiogenesis warrants further investigation and should include a study of VEGF.

Second, we believe that S-MK concentration can become a tool for screening of OSCC. Serum midkine concentrations in patients with early-stage OSCC were significantly higher than those in healthy individuals. This result suggests that S-MK concentration may assist in the early detection of not only OSCC but also other tumours. We measured P-MK concentrations to investigate whether MK leaks from platelets in blood coagulation and an anticoagulant have an influence on the expression of MK. In P-MK concentrations, those values were more elevated in OSCC patients than in healthy volunteers as did in S-MK concentrations. In addition, P-MK concentrations in OSCC patients in the early stage were significantly higher than those in healthy individuals. Platelets and leukocytes in OSCC patients did not relate to the expression of MK. These results suggest that P-MK and S-MK concentrations are useful for early diagnosis of oral cancer. However, concerning the collection of plasma samples, we could not collect those for a long period. As the collected samples were all within 4 years after the surgery, we could not calculate the 5-year survival rate. In reports on other tumours, such as neuroblastoma (Ikematsu *et al*, 2003), oesophageal cancer (Shimada *et al*, 2003), and gastric cancer (Obata *et al*, 2005), serum or plasma MK concentrations were significantly elevated. Because MK is a plasma protein, serum or plasma MK concentrations can be elevated in various types of cancer. As we demonstrated here, an automated FEIA system may be useful in screening for cancer in a large number of samples in a short time.

In both OSCC patients and healthy subjects, S-MK concentrations older than 60 years were significantly higher compared with those younger than 60 years. These analyses suggest that ageing may influence S-MK concentrations. On the basis of the past reports about MK expression in ageing-related diseases, it is natural to consider that expression of MK is elevated in the presymptomatic stages of diseases, such as asymptomatic cerebral infarction, asymptomatic ischemic heart diseases, and a pathological prestage of Alzheimer's disease.

Figures 4 and 5 demonstrated, by means of immunohistochemistry and mRNA *MK* analysis, that MK protein was overexpressed in OSCC tissues compared with healthy tissue from volunteers. However, *MK* mRNA expression in OSCC tissue specimens was not significantly associated with S-MK concentrations. Serum midkine concentrations were previously reported to be associated with MK protein expression in oesophageal cancer cells (Shimada *et al*, 2003), and the 5-year survival rate of a group with high MK expression in OSCC tissue was lower than that of a group with low MK expression (Ruan *et al*, 2007). These results suggest that MK overexpression in OSCC tissues may promote high concentrations of S-MK. Expression of MK may be localised in cancer tissues, because RNA was extracted from some cancer tissues. However, we found no relationship between tumour size and S-MK concentrations and no correlation between *MK* mRNA expression and 5-year survival rates (data not shown).

Expression of a truncated form of *MK (t-MK)* mRNA, which lacks exon 3 encoding the N terminus, has been well documented in various tumours, including colon (Miyashiro *et al*, 1996), breast (Miyashiro *et al*, 1997), gastric (Aridome *et al*, 1998), and liver and kidney (Tao *et al*, 2007). Therefore, we immunostained OSCC tissues with IP-14 antibody, which had an epitope in the C-terminus, and IP-10 antibody, which had an epitope in the N-terminus. One might expect that IP-14, which recognised both the full-length and truncated forms of MK, would show a stronger reaction in carcinoma than IP-10, which did not cross-react with t-MK. However, our results did not support this expectation. IP-14 reactivity was weaker than IP-10 reactivity (Figure 5). Our result agrees with the report of Nobata and colleagues (Nobata *et al*, 2005) that the C-terminus can easily be affected by protease and that t-MK antibody has a low affinity for the C-terminus. Moreover, we first believed that IP-10 would strongly react with MK functioning in protection of the N-terminal part of the molecule from proteolytic degradation (Matsuda *et al*, 1996). However, glandular ducts stained only with IP-14 antibody. This finding indicates that t-MK may be expressed in normal salivary glands, although previous studies reported that t-MK was not detected in noncancerous tissues. Studies to clarify the localisation of t-MK are in progress. On the basis of these results, we analysed S-MK concentrations by using SC2 and SC4 antibodies, which had epitopes in the N-terminus, as does the IP-10 antibody. Even though these antibodies did not react with t-MK, they could detect full-length MK, and the sandwich ELISA performed with these two antibodies was specific and useful for cancer screening and predicting the prognosis of OSCC patients.

In conclusion, S-MK concentrations in OSCC patients were associated with prognosis (5-year survival) but not with conventional prognostic factors such as clinical stage, tumour size, and cervical lymph node metastasis. Midkine expression in blood and cancer tissues is indicative of a strong relationship with malignant potential, and high MK expression suggests a bad prognosis. These studies may lead to trials to inhibit MK expression by using MK antibody and small interfering RNA. The study of MK may elucidate problems of anticancer drug resistance in various cancers and contribute to improvement in prognosis.

## Figures and Tables

**Table 1:** Characteristics and S-MK concentrations for healthy volunteers and OSCC patients

	**Healthy volunteers**			**OSCC patient**			
**Clinical parameters**	**Number**	**Mean±s.d. S-MK concentrations (pg ml^−1^)**	***P*-value**	**Number**	**Mean±s.d. S-MK concentrations (pg ml^−1^)**	***P*-value**	***P*-value**
Mean S-MK concentration	134	419.1±97.9		60	885.9±465.0		<0.001
							
*Sex*							
Male	73	407.0±21.4	0.173	37	949.9±514.7	0.178	<0.001
Female	61	433.5±98.1		23	782.8±358.3		<0.001
							
Mean age±s.d.		63.8±11.0			66.8±11.4		0.075
*Age (years)*							
<60	45	345.3±93.6	0.006	15	645.9±160.8	0.020	<0.001
⩾60	89	456.3±119.2		45	965.8±505.5		<0.001
							
*Smoker*		20.1%			26.7%		0.318
No-smoker	134	418.2±103.0	0.831	44	851.0±450.1	0.340	<0.001
Smoker	27	422.7±75.8		16	981.8±506.5		<0.001

OSCC=oral squamous cell carcinoma; S-MK=serum midkine.

**Table 2:** Relationship between clinical parameters and S-MK concentrations or 5-year survival rate in patients with OSCC

**Clinical parameters**	**No. of patients**	**Mean±s.d. S-MK concentrations (pg ml^−1^)**	***P*-value**	**5-year survival rate (%)**	***P-*value**
*Sex*					
Male	37	949.9±514.7	0.178	71.9	0.141
Female	23	782.8±358.3		56.7	
					
*Age (years)*					
<60	15	645.9±160.8	0.020	78.3	0.449
⩾60	45	965.8±505.5		61.9	
					
*Clinical stage*					
I	7	931.2±443.3	0.977	100	0.003
II	17	864.8±559.7		92.3	
III	7	839.8±317.0		66.7	
IV	29	900.5±464.2		44.5	
					
*Tumour size (mm)*					
<40	35	845.8±461.7	0.434	85.2	<0.001
⩾40	25	942.0±473.2		41.5	
					
*Cervical lymph node metastasis (N factor)*					
N(−)	35	900.1±523.8	0.782	82.5	<0.001
N(+)	25	865.9±377.1		42.4	
					
*Differentiation of OSCC*					
Well	36	822.4±453.2		80.2	
Moderately	17	943.0±367.2	0.327	42.1	0.057
Poorly	7	1095.1±697.7		51.4	
					
*S-MK concentration*					
<650	20	—		82.9	0.045
⩾650	40			56.6	

OSCC=oral squamous cell carcinoma; S-MK=serum midkine.
